# Double Penis—Absent Anus

**Published:** 1893-02

**Authors:** 


DOUBLE PENIS—ABSENT ANUS.—Dr. J. F. Pratt ("Medical Sum.") of Binghampton, New York, reports the following case: A II-para was delivered of a child which above the umbilicus was normal. Encircling that point was an opaque membrane which resembled the peritoneum. It was about two inches in diameter, and terminated rather abruptly in healthy skin. From the right hypochondriac region emerged the transverse colon, and crossed the abdomen externally to the left hypochondriac region, where it entered the abdominal cavity. In the center of the abdomen, and just above the pubic arch, emerged the rectum, which curled up in the direction of the umbilicus, forming nearly one-half of a circle about one and one-half inches in diameter, where it terminated with a circular opening, without a sphincter. There was no trace of an anus; the skin covering the site was perfectly normal and smooth. In either groin was a scrotum and penis. The one on the right side was more fully developed; through it the urine passed. The one on the left was a little smaller, and through the penis, at intervals, blood passed. The thighs sprang from the body at nearly right angles, which was the occasion of the obstruction during labor. From the knees to the ankles the legs passed downwards and inwards nearly to a line drawn through the center of the body. There was double talipes varus and spina bifida. The child lived six days. Although it did not nurse, it swallowed naturally, and was fed at regular intervals; but most of the nourishment passed directly through undigested and in consequence it died from inanition. The skin around the umbilical region took on healthy granulations, and healed about one-fourth of an inch in the direction of the umbilicus. The portion of the bowels that was external to the abdomen was granular in appearance, and remained unchanged.—*Medical Standard.*

